# A systemic framework of energy efficiency in schools: experiences from six European countries

**DOI:** 10.1007/s12053-023-10099-4

**Published:** 2023-03-16

**Authors:** Dmitry Brychkov, Gary Goggins, Edelle Doherty, Natalia Romero, Nadine Roudil, Antonella Di Trani, Abhigyan Singh, Sander Smit, Eilish McLoughlin, Raquel de Castro Rodrigues Lima, Suzan Marie Günbay, Branca Arthur Delmonte, Achim Hill, Christine Domegan, Eoghan Clifford

**Affiliations:** 1 J.E. Cairnes School of Business and Economics, University of Galway, University Road, Galway, H91 CF50 Ireland; 2 School of Geography, Archaeology and Irish Studies, University of Galway, University Road, Galway, H91 TK33 Ireland; 3 School of Engineering, University of Galway, University Road, Galway, H91 TK33 Ireland; 4 Faculty of Industrial Design Engineering, Delft University of Technology, 2628 CE Delft, The Netherlands; 5 Sociology, UMR CNRS, LAVUE, National School of Architecture of Paris-Val de Seine, 3-5 Quai Panhard Et Levassor, 75013 Paris, France; 6 Anthropology, UMR CNRS, LAVUE, National School of Architecture of Paris-Val de Seine, 3-5 Quai Panhard Et Levassor, 75013 Paris, France; 7 R2M Solution, 66 Fenwick Close, Woking, GU21 3BZ UK; 8 School of Physical Sciences and Centre for the Advancement of STEM Teaching and Learning, Dublin City University, Dublin 9, D09 W6Y4 Ireland; 9 School of Engineering, College of Science and Engineering, University of Galway, University Road, Galway, H91 TK33 Ireland; 10 Department of Engineering, Faculty of Science, Technology and Medicine, University of Luxembourg, 6, Rue Richard Coudenhove-Kalergi, L-1359 Esch-sur-Alzette, Luxembourg; 11 Fachbereich Bauen+LebenFachrichtung Bauingenieurwesen, Trier University of Applied Sciences, 54293 Schneidershof, Trier, Germany

**Keywords:** Energy efficiency, School, Systemic approach, Energy literacy, Digital transformation, Collaboration

## Abstract

Schools are complex physical and social institutions within national education systems. They account for significant energy consumption and like other buildings can demonstrate inefficient patterns of energy use. Poor energy performance of educational facilities is an intricate issue driven by complex causality of interconnected and dynamic factors. Addressing this issue requires a systemic approach, which is heretofore lacking. The aim of this research is to present and describe a systemic framework to facilitate energy reduction in schools across different European contexts. This transdisciplinary approach to sustainable energy use has been piloted in 13 post-primary schools located in six countries in northwest Europe. The research implements a series of planned activities and interventions, which help to unveil a systemic approach to improving energy efficiency in schools. The findings demonstrate how this approach, together with its ensuing methodologies and strategies, can contribute to reducing carbon emissions and improve knowledge and awareness around sustainable energy.

## Introduction

Meeting national and international climate targets requires multi-dimensional approaches involving actors across all levels of society (Jensen et al., 2019). Schools and other educational institutions are complex organizations that play a significant role in framing the climate challenge and shaping our individual and collective response to achieving necessary reductions in energy use (Meehan et al., 2018). According to some sources, education systems account for rather modest shares of the total energy consumption at the regional or national level (Chung & Yeung, 2020; Dascalaki & Sermpetzoglou, 2011). Other sources name schools as “significant energy consumers – on a par with residential and office buildings” (Thewes et al., 2014, p. 469), emphasizing their tangible share of carbon emissions in the public sector (Schwartz et al., 2021). Irrespective of the actual contribution of school buildings to the energy consumption balance, educational facilities play an important role as social institutions (AlFaris et al., 2016). They can shape students’ views, beliefs, and attitudes, as well as equip them with the knowledge and skills that impact their careers and lifestyle choices. Schools also have a spillover effect on wider society manifesting from mutual relationships emerging between people and their social contexts (Ashwin et al., 2015). As such, “school buildings can contribute a *significant positive culture of energy efficiency*” (Chung & Yeung, 2020, p. 2, emphasis added). In general, educational establishments could be regarded as vital arenas of social and environmental change within the quadruple and quintuple innovation helix frameworks, which link environmental issues, academic research, policy making, society, and business (Morawska-Jancelewicz, 2022). This goes in line with the holistic character of all sustainable development goals (SDGs), where such global goals as affordable and clean energy and quality education, as well as health and wellbeing, are closely interrelated (Hoque et al., 2022; UNICEF, 2019).

Nonetheless, this potential contribution is impacted by complexities in (i) social interactions and behavior change in a multi-stakeholder environment and (ii) physical interactions and building infrastructure management. Since energy efficiency and energy management are socio-technical concerns which embody technological, human, and interactionist issues, these issues must be addressed in an integrated and systemic manner (Bernardo et al., 2018; Goggins et al., 2019; Zhang & Bluyssen, 2021). However, the lack of (low-cost) systemic approaches (or frameworks) to energy efficiency in a complex multi-stakeholder social and physical environment of schools is a significant gap.

The aim of this research is to present and describe a systemic approach to addressing energy efficiency in schools, which can be applied across different educational systems and contextual conditions. This approach is developed within a European project entitled ENERGE. ENERGE aims to reduce energy use in 13 secondary schools in six northwestern European countries and to transfer the obtained experience to other schools as a viable integrated solution. The project comprises a series of planned activities and interventions, which help to structure a conceptual framework for energy reduction in schools. The overall contribution of this paper lies in demonstrating how a systemic approach to energy efficiency in schools can be realized and generate benefits for schools and wider society.

The remainder of this paper is structured as follows. In the “Literature review” section, we present a review of related literature, including a focus on systemic approaches to sustainable energy use in schools. In the “Methods” section, we provide a detailed explanation of the employed methodology, while the “Results” and “Discussion” sections report and discuss relevant framework-based results and visualize the framework. The paper concludes by outlining the benefits and innovative nature of the framework.

## Literature review

### Approaches to energy efficiency in schools

Energy efficiency in educational institutions is an important area of academic research, policy, and practice (Rospi et al., 2017). A significant number of studies are dedicated to various strategies to improve energy efficiency of educational facilities, incorporating both technical measures and social changes (Lourenço et al., 2014). According to Rospi et al. (2017), studies addressing energy use in schools may involve (a) in situ investigation of (perceived and measured) indoor environmental quality (IEQ), including such parameters as temperature, humidity, illuminance, CO_2_ concentration, etc. (e.g., Zhang & Bluyssen, 2021); (b) assessment of school buildings and their energy-related characteristics, in interaction with the outside physical environment, via energy audits (e.g., Arambula Lara et al., 2015) and with the help of benchmarks (e.g., Thewes et al., 2014); and (c) surveys of the school building inhabitants to analyze their thermal (and other) sensations and preferences, as well as their adaptive behaviors (e.g., Korsavi et al., 2020). The latter imply various (thermal, visual, acoustic, etc.) questionnaire-based indoor comfort studies (Fuentes et al., 2008), including post-occupancy evaluations in various types of educational facilities across different geographic locations (see Jowkar et al., 2020 for a comprehensive list of such studies). There are also examples of detailed assessments of school building conditions and design, including human comfort, as well as various physical, social, psychological, cognitive, and other aspects of learning environments (Imms & Byers, 2017; Roudil et al., 2015; Sanoff, 2001).

The capacity for energy efficiency in the school environment can be significantly diminished by the sheer intricacy of user behavior in a multi-stakeholder environment (McKenzie-Mohr, 2020; Stuart & Ozawa-Meida, 2020). Within school building energy management, Bernardo et al. (2018) single out the following key stakeholders: (a) the schools themselves (with their own complex administrative and management structure and regulation, including members of the school board of directors, students, and staff, as well as the parents associations), (b) the school management companies, (c) facilities management companies, (d) energy supply companies, (e) energy services companies, (f) energy-related equipment manufacturers and retailers, (g) regional/national governments and international institutions, and (h) local communities. With respect to the day-to-day operation and maintenance of the building infrastructure, Stuart and Ozawa-Meida, (2020) suggest categorizing such stakeholders into the three groups: (1) building users (e.g., school students and staff); (2) energy professionals (e.g., energy supply and services companies), who are responsible for energy maintenance and relevant infrastructure operation; and (3) strategic managers (e.g., school board of directors), responsible for the strategic management of infrastructure.

All these stakeholders are engaged in complex feedback relationships, which underlie social interactions and are predicated on specific contextual influences, internal structures, and hard-to-measure variables (Bernardo et al., 2018). Such complex interactions make behavior change a very difficult task. The difficulty of behavior change is augmented by the complex nature of social interactions within the education systems, including insufficient level of students’ and teachers’ self-efficacy (Mathwasa & Sibanda, 2020; Tipon et al., 2021), contested social norms (Schneider, 2000), and inertia and path dependence (Thomas, 2002). Students demonstrate lack of control over energy-saving activities in school buildings, while teachers often have limited scope, information, and incentive to carry out these activities (Zhang & Bluyssen, 2021). Indeed, these are typical issues for the public sector in general, which can demonstrate free-riding consumer behavior to energy saving (Stuart & Ozawa-Meida, 2020).

Similarly, the impact of the physical school environment on energy efficiency involves the complexity of energy infrastructure and its management, as well as its intricate interactions with outside environment and other system elements. School buildings are often heterogeneous with variations in age and complexity of technology and energy infrastructure, and differences in its management and maintenance (Stuart & Ozawa-Meida, 2020). Managing dated building stock is difficult due to the investment-intensive character of the stock and a possibility of disrupting vital educational activities. Large-scale infrastructural projects targeted at thermal renovation of building envelopes may not be economically beneficial and cost-effective subject to existing electricity prices and building construction costs (Friedman et al., 2014). Moreover, new or renovated school building stock has been associated with increased electricity consumption (Thewes et al., 2014) and rebound effects such as increased indoor temperatures (Sorrell & Dimitropoulos, 2008). Therefore, minimizing energy use in schools should be complimented with a focus on the thermal comfort of school occupants, which exists at the intersection of social, psychological, technological, and physical domains (Jowkar et al., 2020; Korsavi et al., 2020). However, the practice of thermal comfort maximization in the real world has always posed a problem, especially in the public sector (Hellwig et al., 2019).

Energy efficiency can be also delivered via the so-called Energy Service Companies (ESCOs) and Energy Performance Contracting (EPC). These approaches are realized as integrated and holistic mechanisms, which merge various aspects of energy efficiency, including financial support, technical knowledge, management solutions, market research, engagement tools, and communication skills (Marino et al., 2010). There are cases of ESCO application in schools, when students become active members of energy efficiency management in a school-learning environment (Trombley, 2016). Among key barriers for ESCO approach manifestation, Bertoldi and Boza-Kiss (2017) name issues with procurement, legal impediments, lack of facilitation, and financial costs.

### Energy literacy and digital transformation for learning purposes

The term literacy in an educational context can be defined as the capacity of students to apply knowledge and skills in key subject areas and further to be able to analyze, reason, and communicate effectively as they pose, solve, and interpret problems in a variety of situations (UNESCO, 2006). Energy literacy is an iteration of literacy that is viewed as an essential tool which sensitizes citizens to create sustainable energy consumption habits (Martins et al., 2020). Energy literacy also shares characteristics and learning objectives in the domains of science and technology literacy, which incorporate dimensions of knowledge, attitude, skills, behavior, and civic engagement. According to DeWaters and Powers (2013), the energy literate student should (a) have a basic understanding of science and how energy is used in everyday life; (b) understand the impact that energy production and consumption have on all spheres of our environment and society; (c) be sensitive to the need for energy conservation and the need to develop alternatives to fossil fuel-based energy resources; (d) be cognizant of the impact of personal energy-related decisions and actions on the global community; and (e) strive to make choices and decisions that reflect these attitudes with respect to energy resource development and energy consumption. Dwyer (2011) described energy literacy more simply as a baseline fluency and knowledge of complexities related to energy use. As such, energy education should impact knowledge, skills, attitudes, values, decisions, and actions (Barrue & Albe, 2013; DeWaters & Powers, 2013).

Energy education is connected to digital learning and the use of digitization, since digital transformation is affecting the way people learn and live. Digital transformation also deeply impacts the teaching profession. In the recently published EU Digital Education Action Plan (2021–2027), it is stated that the digital transformation has transformed society and the economy, but until the Covid-19 crisis, the impact of this transformation on education was much more limited (European Commission, 2021). A study found that on average less than 40% of educators across the EU felt ready to use digital technologies in teaching (European Commission, 2021), while OECD data shows that, prior to 2018, on average 25% of the teachers and students did not use digital devices for teaching and learning (OECD, 2021). Large disparities between regions and countries exist in terms of digital infrastructure use as fewer than 1 out of 5 students attending schools have access to high-speed Internet in some countries (European Commission/Deloitte/Ipsos MORI, 2019). The majority of students use digital devices for browsing the Internet for schoolwork, emails, and online chatting, but, prior to the Covid-19 pandemic, a much smaller number of students applied digital devices for using learning apps or playing simulations at school (OECD, 2021).

### Systemic approaches to sustainable energy use in schools

Several examples of literature document *systemic* approaches to the issue of energy efficiency in schools. These holistic approaches feature some basic commonality of treating the issue of energy efficiency from multiple perspectives (technological, social, personal, economic, ecological, etc.), involving recognition of the fact that to tackle the issue of energy efficiency in schools, one has to deal with so-called wicked problems. These are characterized by uncertainty of explanations sensitive to the point of view, ever-changing and contingent nature of problems themselves, and intricate and ambiguous feedback causality, as well as the difficulty of finding solutions, often causing adverse side-effects (Rittel & Webber, 1973; Jackson, 2019).

A systemic methodological approach to energy management in schools, which integrates quantitative and qualitative data analysis, has been suggested by Lourenço et al. (2014). Their approach operated at multiple levels of system analysis and addressed environmental, functional, and social factors influencing energy efficiency in schools, including associated energy use patterns, resource sources, building design, behavior change, raising awareness, and knowing building user needs. Among the limitations of their approach, the authors singled out the difficulty of understanding causality of user behaviors and their change.

Dias Pereira et al. (2019) have stressed the importance of combining indoor environmental quality (IEQ) and energy conservation in schools via a holistic approach. Their approach integrated multiple domains. There were as follows: energy auditing, IEQ control, energy efficiency planning, occupancy management, calculation of financial aspects of energy consumption, building management system operation, and some elements of responsible behavior change. However, the latter was not particularly pronounced in contrast to the research by Johansson et al. (2021), where behavioral change at both individual and collective levels is seen as one of the central pathways toward energy efficiency in buildings.

Geraldi and Ghisi (2020, p. 1) have provided a holistic questionnaire framework for assessing the actual energy performance of school buildings, including both physical and human parameters, which “gives way to the development of consistent building stock modelling.” The framework allows monitoring of a rich picture of energy efficiency in schools by asking about such issues as energy management (e.g., awareness of monthly energy consumption), environmental satisfaction, patterns of usage, etc.

A holistic approach to energy management in schools was proposed by AlFaris et al. (2016, p. 799), who stated that “a key strategy to increase the energy efficiency in a systemic way and professional approach is to develop an energy management program.” Their energy management program primarily implied the establishment of a dedicated multi-stakeholder energy management committee responsible for (i) regular energy audits to baseline energy usage (e.g., the historical energy data of the school) and (ii) setting realistic targets and development/implementation of action plans, strategies, and policies, including in such sectors as engineering, economics and economic feasibility, management, etc. The committee comprised a maintenance engineer, management specialists, energy efficiency experts, teachers, and students. The program also included a training element, but key social and cultural drivers of energy efficiency were missing from the program.

In contrast, Pietrapertosa et al. (2021) aimed to increase energy efficiency in public schools by demonstrating the effectiveness of pupils’ engagement to promote energy savings through behavior change. Likewise, Cornelius et al. (2014) put a major focus on theory-informed school-based interventions to promote energy-saving behavior change, rather than simply changing awareness or improving knowledge. They highlighted the importance of relying on widely tested behavior change approaches (including in the domain of energy saving), like community-based social marketing (McKenzie-Mohr & Smith, 1999). Koumoutsos et al. (2015) investigated a combination of a web platform of energy consumption monitoring in schools (IPv6 platform, http://gen6.sch.gr) and social engagement via a school network. In the same vein, Carter (2014) integrated quantitative energy use monitoring by school children with their education and engagement in managing complex energy systems.

A further advancement was the use of soft systems methodology and value-focused thinking by Bernardo et al. (2018) to better understand the multiple aspects that influence energy efficiency of school buildings and incorporate energy and non-energy aspects to provide a more systemic approach to the issue of improving energy efficiency in schools. In order to achieve this, the authors (a) identified key stakeholders affecting energy efficiency in schools, along with their roles and concerns in this process; (b) highlighted the key problems associated with energy management in schools; (c) performed a structured analysis and description of the focal system and systemic analysis of solutions for the system transformation; (d) formulated a conceptual model of the activities within the system; (e) collaboratively debated changes aimed at improving the system; and (f) provided a structured tree of fundamental objectives to correct the system. This approach relies on a participatory methodology and interaction with key system’s stakeholders, involving students, teachers, and support staff.

Despite the wealth of literature on energy efficiency in schools, including some holistic approaches, there is a significant gap in the frameworks above that could really integrate multiple aspects of energy efficiency improvement in schools. There is a dearth of writings on systemic implementation of such integrated frameworks, while some elements of energy efficiency in schools are simply missing or being not adequately addressed. Among these elements, one can mention the following gaps pertinent to energy efficiency in schools: limited focus on energy literacy, non-consistent approach to user behavior, the need to consider personal energy-related experiences and lived histories, absence of viable business plans to realize energy efficiency projects in a continuous manner, and impaired stakeholder networking/relationship building. The integrated methodology to address these gaps is described in the next section, which lists all methods within the ENERGE framework of energy efficiency.

## Methods

### Context analysis

ENERGE stands for “Energizing education to reduce greenhouse gas emissions.” It is an InterReg northwest Europe project, with transdisciplinary partners representing academia, business, non-profit organizations, and regional governmental authorities. A sample of 13 post-primary schools (“the project schools”) in six northwestern European countries, namely France, Germany, Ireland, Luxembourg, the Netherlands, and Northern Ireland, collectively referred to as “the project countries,” was selected by purposive non-probability sampling (Saunders et al., 2019). The selected schools served as experimental laboratories for developing and testing a conceptual framework of energy efficiency in schools (“the ENERGE framework”).

The detailed analysis of the energy use in project schools was based on Zhang and Bluyssen (2021) and Geraldi and Ghisi (2020), with additional information and analysis considered as appropriate. The project schools provided information on the following fields: (1) occupancy patterns; (2) ownership and governance; (3) school layout details; (4) intra-school groups; (5) heating/cooling systems; (6) typical classrooms; and (7) electrical and heating energy consumption by fuel type. The latter point was calculated by dividing the used energy by the gross floor area in square meters as the most traditional way (Jota et al., 2017). It was of particular interest to understand how energy consumption in a project school compared against those of other post-primary schools in the specific region.

It is worth noting that some of the methods have been trialed within the context of specific project countries or project schools. This was done for two reasons. Firstly, the Covid pandemic prevented reaching multiple stakeholders and complicated information gathering. Secondly, the prime objective was to present a systemic framework for raising energy efficiency in schools, rather than to roll out all trialed methodologies to every project school. Indeed, the flexibility of the ENERGE framework and the options it provides were considered attractive by schools. For instance, to understand how energy consumption in a project school compared against other post-primary schools in the region, the schools in Ireland were trialed. In Ireland, the relevant data on energy consumption by post-primary schools was provided by the Sustainable Energy Authority of Ireland (SEAI). The dataset featured 416 post-primary schools and covered a 4-year period from 2016 to 2019. It included data on total school population, total useful floor area (TUFA), and electrical/heating energy use as a 4-year average of total primary energy requirement (TPER).

### Stakeholder analysis

Under a stakeholder analysis, ENERGE researchers assessed the network of stakeholders affecting or affected by the achievement of energy use efficiency within a school system. The ENERGE framework uses the following stakeholder analysis methods: (a) establishment of internal working groups (the ENERGE Committees and Teacher Network); (b) power-versus-interest grid exercise; and (c) stakeholder analysis survey. These methodological tools help to identify and classify key stakeholders who can affect or are affected by the focal problem; assess the stakeholders’ levels of interest and/or power in relation to the focal problem; select, recruit, and engage diverse stakeholders pertinent to the focal problem; and bring together stakeholders from various cross-country contexts (McHugh et al., 2018). The advantage of the ENERGE project within this area lies in (i) its more practical focus on stakeholder analysis via the use of specific methodological tools; (ii) stakeholder engagement via establishment of different school-based working groups with strong educational focus (see the next sub-section); (iii) the collaborative nature of stakeholder interactions; and (iv) the cross-country nature of collaborative efforts.

### Establishment of internal working groups (“ENERGE committees” and Teacher Network)

ENERGE committees in all project schools (one per project school) were formed to include 4–5 students and a teacher. The recruitment of candidates for the ENERGE committees and teacher network was based on volunteering and snowball sampling, when the project partners initially identified a relevant candidate or candidates that later suggested other people ready for the membership (Bryman, 2016). The Teacher Network, including 19 teachers from 11 partner schools across all project countries, discussed and shared ideas on teaching energy topics and developing energy literacy modules and activities. It also built channels for communication and collaboration across schools.

### Power-versus-interest grid method of stakeholder analysis

Power-versus-interest grids determine which players’ interests and power bases must be considered in order to address a focal problem (Bryson, 2004). Subject to matrix interrelationship of power and interest, the grid categorizes stakeholders into 4 groups: crowd (low power, low interest), subjects (low power, high interest), context setters (high power, low interest), and players (high power, high interest). The main objective of the exercise was to engage the ENERGE committee members in *identifying and classifying key stakeholders related to energy use* in post-primary schools and homes, by placing these stakeholders on the power-vs-interest grid and then substantiating their choices. The exercise was tested in Ireland with 14-year-old students (*n* = 4) and a teacher (the exercise was facilitated by two project partners). The exercise was also undertaken by project partners (*n* = 16). An initial long list of possible stakeholders was drawn up, with a total of 31 identified, grouped under 6 categories, focusing on external (non-school) stakeholders.

### Stakeholder analysis survey

A survey was held among the school personnel of 10 project schools within the period from April 2020 to June 2020. The survey obtained information on (a) stakeholder roles and interactions within the school system; (b) engagement in energy-related activities within the school; (c) levels of control over energy-related activities within the school; and (d) commonality of performing energy-related activities within the school, including those related to knowledge and attitudes. The number of responses was 166.

### ENERGE Digital Platform to monitor electricity and indoor environmental quality

To monitor electrical energy consumption and indoor environmental quality (IEQ), the ENERGE Digital Platform was developed and installed in all project schools. The platform comprised (a) two meters to monitor and transmit data on electrical energy consumption and (b) four indoor climate sensors to monitor and transmit data on temperature, humidity, carbon dioxide, lighting, and noise levels at specific school locations. Inspection visits to the project schools were made to select meter/sensor installation sites and identify the existing circuitry configuration of electrical distribution boards. The devices regularly transmitted data for analysis, which was subsequently linked to the development of energy literacy modules (see the next sub-section).

### Development of energy literacy modules

ENERGE implements energy-related education in the curriculum across the project countries to improve energy literacy among second-level students (DeWaters & Powers, 2011). The ENERGE energy literacy model was developed to identify energy literacy characteristics and inform the development of ENERGE teaching and learning activities. A database of energy-related teaching/learning activities was compiled and shared with Teacher Network members. In parallel, a systematic literature review was carried out to address (a) energy literacy definitions; (b) knowledge, skills, attitudes, or values associated with energy literacy; (c) conceptual frameworks for energy literacy; (d) principles of energy literacy learning/teaching; and (e) pedagogical practices for developing energy literacy.

Further, the Teacher Network members provided feedback on the alignment of the ENERGE energy literacy model with their subject curricula, as well as contributed to the database expansion by suggesting relevant energy literacy units and activities. The scope and content of energy-related modules, units, and activities were agreed upon, and the Teacher Network members started to pilot them among their students. The original units/activities were reviewed and finalized based on feedback provided by teachers after piloting in their classrooms.

### Interviews

Key informant interviews with principals (*n* = 4) and head teachers (*n* = 2) were held in schools in Ireland and Northern Ireland. The interviewees were selected by purposive non-probability sampling to represent these schools. The interviews addressed the following key topics: (1) implementation of energy efficiency measures; (2) the decision-making process with respect to energy efficiency; (3) current and historic expenditures on energy efficiency; (4) the use of educational materials to teach energy efficiency and/or sustainability; (5) availability of strategies and relevant knowledge on energy efficiency; (6) using schools as sustainability-teaching labs; (7) expectations from the ENERGE project; and (8) the unique selling proposition of the ENERGE project.

The ENERGE framework also includes other research methods including a survey to analyze attitudes and behaviors of students in relation to energy efficiency in their schools; indoor climate comfort survey among the students to analyze their comfort perceptions and preferences; the literature-based analysis of national education systems of the project countries; and the development of future ENERGE business scenarios. The methodologies and results of these elements are not addressed in the paper since their implementation is still ongoing. However, all elements of the ENERGE methodology, including context analysis, stakeholder analysis, networking and stakeholder engagement, surveys, building audit methodology, ENERGE digital platform installation, key performance indicators, education system governance analysis, energy literacy module development, and in-depth interviews, were presented as finalized reports ready to be communicated to all interested stakeholders, and specifically to school management. The corpus of these ENERGE reports, available to the public, experts, and all interested stakeholders, forms and solidifies the basis of the project methodology.

## Results

### Context analysis

Context analysis was a starting point for establishing a baseline to monitoring progress in energy efficiency over the course of the project. This analysis was holistic, moving from an overview of specific components with their basic characteristics to understanding the whole system and environment (Crawley & Aho, 1999). As could be expected, the analysis revealed large variability in the selected project schools with respect to multiple parameters including characteristics of physical localities, curriculum orientation; occupation characteristics; operational patterns; management approaches; building and energy use elements and typologies. Such diversity is also a reflection of the variability of the national education systems, which vary widely in terms of structure and curricular content (UNESCO Institute for Statistics n.d.). The national education systems of different European countries also feature diverse approaches with respect to the way funds and other deliverables are distributed (European Commission/EACEA/Eurydice, 2019; Pedreschi, 2020). Despite these differences, the ENERGE project aims to develop a generalized framework (though adaptable for specific conditions) for achieving energy efficiency that can be applicable across diverse national and regional contexts.

The building stock across the project schools varied greatly in many different aspects including heating provision (local boilers using gas, kerosene or woodchip, use of district heating, etc.), building fabric (stonework, blockwork, prefabricated units, single-glazed windows, triple-glazed windows, etc.), and other aspects. The curriculum orientation type of the project schools included general/classic (e.g., Ireland, Northern Ireland, the Netherlands), technically oriented (e.g., Luxembourg), agricultural (e.g., France), and scientific (e.g., Germany). There was also presence of the vocational element (e.g., Luxembourg, France, Germany), which meant good acquaintance with technical and energy-related subjects. To account for differences in curricula, interventions aimed to improve energy efficiency should account for the fact that there will be a range of knowledge among the staff and student cohorts.

Ownership structures varied between countries. The Diocese of Galway was stated as the school building owner of Coláiste Éinde (Ireland), while for the second Irish school (Seamount College Kinvara), the owner was the Department of Education. In Luxembourg, all three schools were owned by the Administration des Bâtiments Public or the public building administration, affiliated to the central government. For the French schools, school ownership was at a regional level—at the regional council of Centre-Val de Loire. Ownership rested at a local (city) level with schools in the Netherlands, where schools have large autonomy. In the two Dutch schools, the school owners were vo Haaglanden (the secondary education board in Hague region) and Stichting Boor & Gemeente Rotterdam. In Germany, the city administration of Trier owned Max-Planck-Gymnasium Trier gymnasium and the district administration of Eifelkreis Bitburg-Prüm owned Berufsbildende Schule Prüm. In all project schools, budget allocation for school maintenance was executed by school owners.

**Fig. 1:**
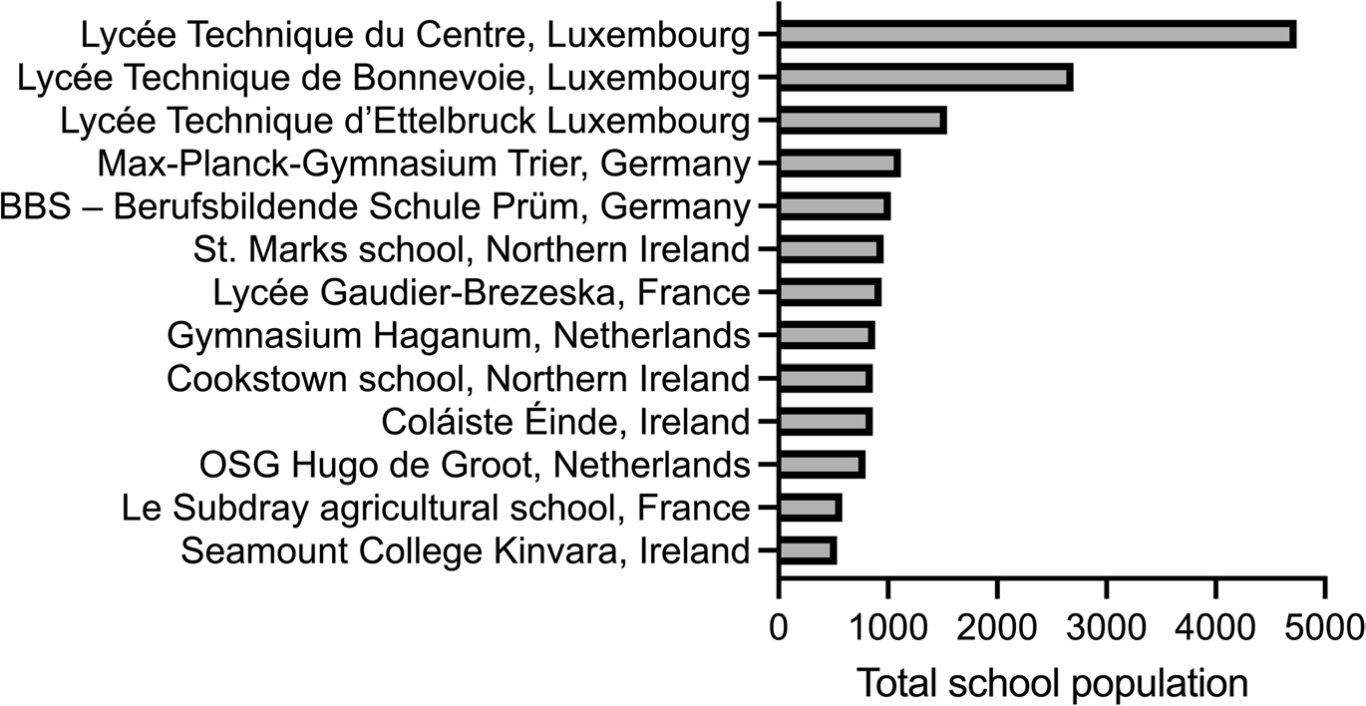
School population in the project schools

The sizes of the project school populations, inclusive of students and staff, varied from 500 to several thousand people (Fig. 1). Interventions which focus on overall change across the school cohort should consider the varying level of effort which may be required to apply an intervention due to different school size levels. Purely quantitative differences in the school population may also lead to differences in school interactions and population segments (for a review of the effects of school size on school practice and outcomes, see Opdenakker & Van Damme, 2007).

**Fig. 2:**
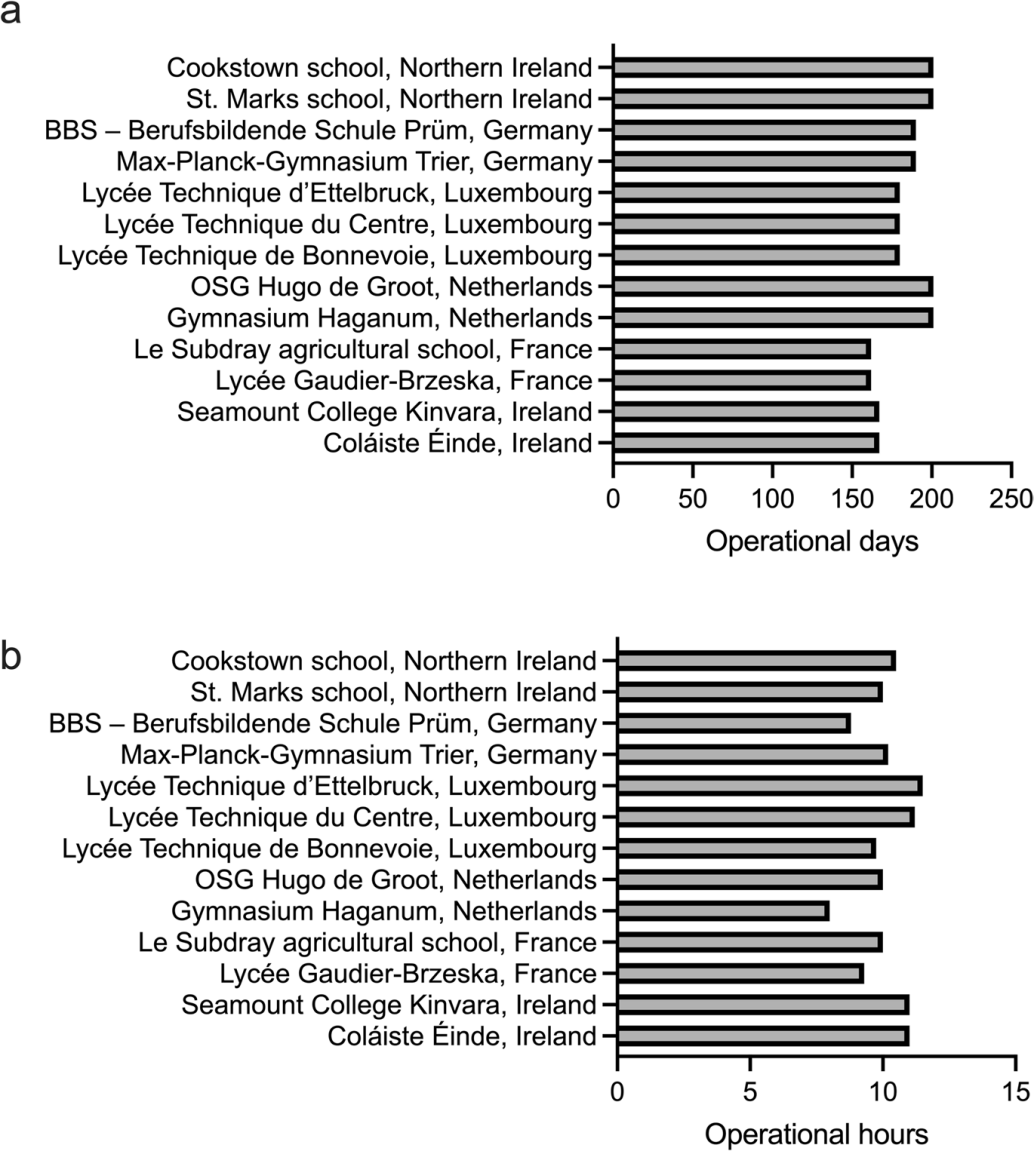
Operational days (**a**) and hours (**b**) in the project schools

The project schools demonstrated a certain level of variance with respect to their operational days and hours (Fig. 2a, b). The term “school operational days” refers to the annual duration of the school operation, starting from the official staff planning event at the beginning of the school year until the end of the school year (when final teaching classes are held), excluding weekend days and recognized holiday periods. It is clear from Fig. 2a that the project schools in France and Ireland have fewer operational days, which could affect energy consumption. However, the difference mainly refers to summertime when energy consumption is generally lower (low/no heating requirement). This is evident from analysis of monthly energy consumption (for both electrical and heating energy) in all project schools over several years. For example, to illustrate this point, energy consumption in the Lycée Gaudier Brzeska (France) in July 2019 was almost 8 times lower than in January 2019. The same pattern applies to all other project schools. This offsets the difference in energy consumption caused by the variation in operational days. The minor influence of operational days can be also confirmed by comparing energy consumption in some project schools. For instance, the Berufsbildende Schule Prüm (Germany), which has less operational days and smaller GIA than the OSG Hugo de Groot (Netherlands), demonstrates higher specific energy consumption.

The term “school operational hours” refers to the number of hours the school remains operational during each working day, starting from the morning arrival of the first personnel to the time all staff and students have left the school, but does not include boarding school activities (i.e., late-evening/night operation). Actual school operational hours might feature very diverse and complex patterns associated with mixed use of school buildings, including activities of after-school clubs and volunteer organizations, parent meetings, school theatrical performances, etc. These complex operational patterns mean that any energy efficiency measures focusing on out-of-hours energy checks should accommodate these differences.

The context analysis revealed various dichotomies in educational contexts of the project countries, like levels of centralization/control vs levels of autonomy/independence. For instance, despite a general trend for increasing school autonomy in Europe, there are still significant differences between countries. While some countries (e.g., Netherlands, Estonia, Finland) grant a high degree of autonomy to secondary schools for managing financial and human resources, in other countries (e.g., Greece, Cyprus, Malta, Turkey), schools have very limited or no freedom in this area (OECD, 2013). This variation between countries is dynamic both at temporal (i.e., could vary over time) and geographic levels (i.e., subject on specific location). Therefore, the project schools featured a varying level of managerial autonomy. For instance, the Irish schools (or their management boards) and Gymnasium Haganum (Netherlands) are responsible for their own maintenance. For the French, German, and Luxembourgish schools and one Dutch school (OSG Hugo de Groot), the responsibility for the maintenance of their schools lies with specialized technical agencies. These diverse maintenance patterns can affect the applicability of both managerial and technical interventions; some schools may not be able to easily adjust maintenance, operational, or managerial procedures where inefficiencies are detected.

The project schools present with varying annual energy consumption values due to characteristics which are unique to each school. This is not an uncommon result when comparing energy consumption or efficiency across various buildings; to tackle this, there are many ways in which to report energy consumption figures. For instance, variables which can affect energy efficiency key performance indicators (KPIs) include floor area (as detailed above); occupancy rates; occupancy hours per year; building use; building characteristics such as construction, age, and orientation; external weather (typically corrected using degree days (DD)); and more. The authors of this paper have spent considerable time focusing on the selection of appropriate KPIs (which will be presented in a separate paper due to the complex nature of the topic).

**Fig. 3:**
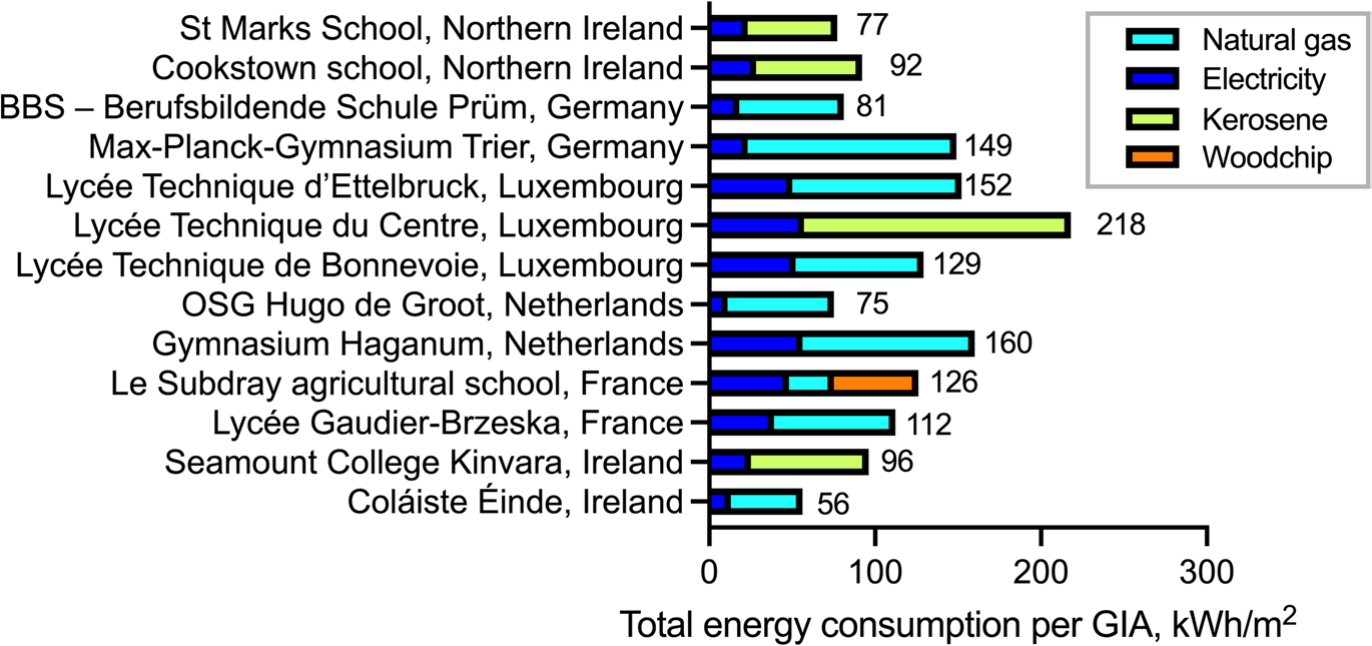
Annual total consumption of energy (electrical and heating energy) per square meter of gross internal area (GIA) in the project schools by energy and fuel type. GIA is defined as the total useable school floor area (classrooms, storerooms, utility rooms, inside walls, corridors, etc.), measured to the internal face of the perimeter (outside) walls of school building(s), excluding these outside walls

For the purpose of this paper, energy efficiency is reported based on the gross internal area of each school (Fig. 3). With respect to total annual energy consumption per GIA, the highest value of this parameter was observed at the Lycee Technique du Centre (Luxembourg) and the lowest at the Coláiste Éinde (Ireland). Lycée Technique du Centre (LTC) provides an interesting case study in terms of relatively high energy consumption. This could be attributed to several factors. Each year, LTC hosts 3600 students and apprentices with more than 800 candidates enrolled in adult courses, all supervised by 340 teachers (Da Cruz Antunes, 2019). This provides the largest school population and the highest student-to-staff ratio among all project schools. This is within a GIA of 23,971 m^2^—similar to the GIA of Lycée Gaudier-Brzeska (France), which has a much smaller school population. The main building dates to 1965 and was modestly renovated in 1985. The school building structure is divided into three different blocks and six departments. Since the school specializes in such areas as engineering, mechanics, optics, and numerous fields of professional training, all departments have a lot of energy-intensive facilities, like workshop rooms, IT sections, laboratories, chemistry sections, etc. Significantly, the school also has a swimming pool, a large sports center, and a festival hall. The electrical system caters for lighting, circulating pumps, ventilation, cooking and kitchen appliances, IT equipment, and large workshop machines. The lighting system comprises thin fluorescent tubes with the following power specifications: 2 × 49 W tubes for workshops, 4 × 18 W tubes for classrooms, and 4 × 28 W tubes for corridors (there are 117 available rooms in the school). These lighting devices are often not able to respond to presence detectors. Three large areas of the school are ventilated, including the swimming pool (with a volume flow of 9500 m^3^/h), the workshops (with a volume flow of 14,000 m^3^/h), and the festival hall (with a volume flow of 15,000 m^3^/h). The LTC heating system uses three large fuel oil tanks with a total volume of 90,000 L. In addition, three fuel oil combustion plants operate in the school, two with a maximum output of 1400 kW each and one with 1970 kW. All premises are heated by radiators with valves positioned in fixed control positions. Therefore, the combination of these factors, i.e., a modestly renovated building, a significant school population and relatively smaller GIA (as compared to other project schools), abundance of energy-intensive facilities (e.g., a swimming pool) and dated energy systems, provides the high level of energy consumption in this school. This example also illustrates how the interdependence of such factors could determine the large diversity of energy consumption values among the project schools. It is pertinent to mention here that some of the factors influencing school building energy consumption may not render a direct impact on such consumption. For example, according to Hoos et al. (2016), the final heat energy consumption in schools does not directly correlate with school building age due to subsequent partial or full renovation.

**Fig. 4:**
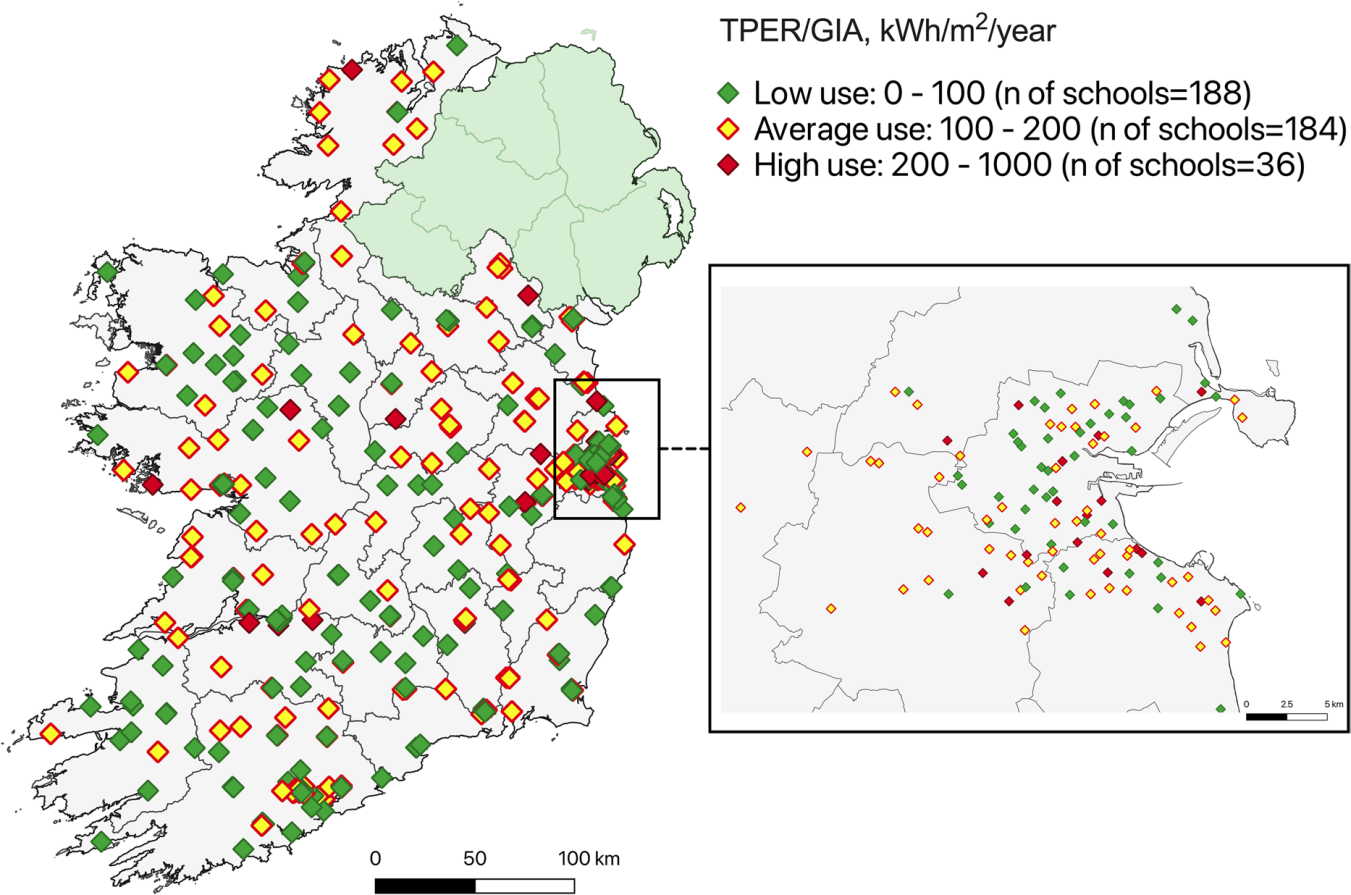
Energy consumption in 408 Irish schools

As discussed in the “Methods” section, it was of interest to compare at least some of the project schools with other corresponding schools at the national level. In order to perform this, the project partners obtained a dataset on energy consumption in over 400 Irish schools (Fig. 4). Energy consumption was measured as a ratio (kWh/m^2^/year) of total primary energy requirement (TPER) to gross internal area (GIA). These schools were segmented into the following three categories: (a) schools with low use of energy; (b) schools with medium use of energy; and (c) schools with high use of energy. This categorization was based on the research by Hoos et al. (2016). The mean value of energy use for this sample was about 120 kWh/m^2^/year. The analysis reveals that the bulk of the sampled schools in Ireland is within low- (*n* = 188) and average-energy users (*n* = 184), which nevertheless may mean that they could benefit from various energy efficiency measures.

With respect to school maintenance and infrastructural operational issues, as well as the greatest challenges/barriers to their resolution, the project schools primarily noted the following issues:

- Financing issues (a lack of financing; a difficulty of seeking funding from national institutions; incremental character of financing, which arrives in installments and thus covers only specific areas)
- Listed property type of building
- Finding proper entities who perform infrastructural maintenance in schools

Some schools reported a lack of substantial sustainability-related programs, or that such programs could be sporadic and involve small initiatives, such as reduction in plastic use and teaching sustainability in chemistry/physics classes (e.g., telling students to bring their own bottles). Comfort and thermal comfort surveys were not commonly found to occur in the project schools, and when they did, they were not applied consistently. For instance, one school management board conducts a survey every 2 years. This survey includes a question about how students rate the indoor climate on the scale from 1 to 5. However, the survey did not ask about (thermal) comfort in buildings directly, but rather about the general social atmosphere. There were also issues with electricity use measurements and controls (e.g., fragmented character of reporting; metering logistics issues; issues connected with the change in energy suppliers and interrelationships with them; etc.).

### Power-versus-interest grid method of stakeholder analysis

**Fig. 5:**
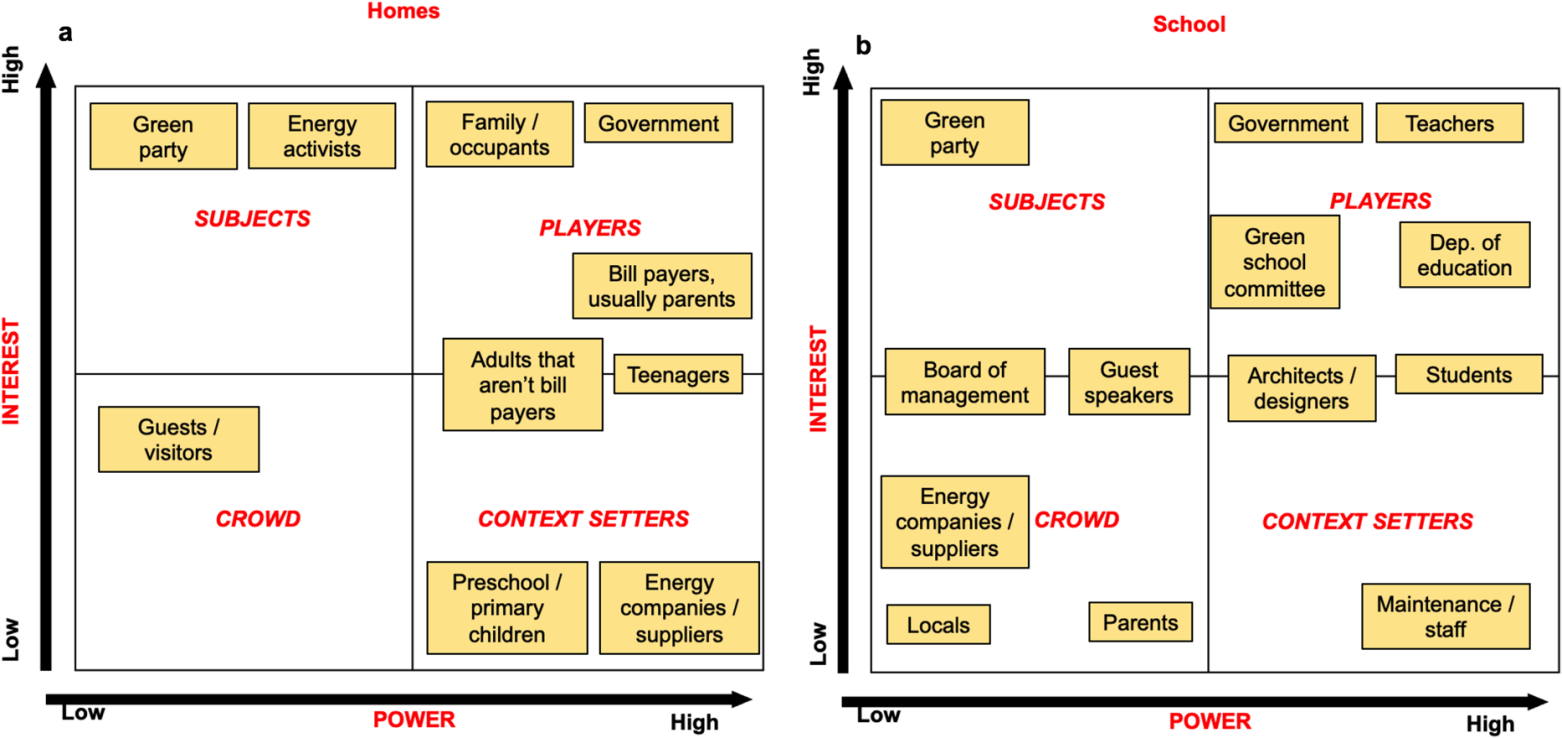
Power-vs-interest mapping of stakeholders related to energy use at home (**a**) and school (**b**) by members of the ENERGE Committee. Some stakeholders are viewed as occupying transboundary positions

Using the methodology of the power-vs-interest grid exercise, the members of the ENERGE Committee mapped the stakeholders, related to energy use at their homes (Fig. 5a) and their schools (Fig. 5b). The power-vs-interest grid exercise for stakeholder classification helped to improve the students’ capacity for mental modeling in comparison with simply brainstorming lists of possible stakeholders. It identified new stakeholders (e.g., guest speakers in schools, green school committee) and stimulated students’ ability to recognize complex relationships between stakeholders. Members of the ENERGE Committee were able to see a variability inside bigger groups, thus segmenting larger groups of stakeholders into separate stakeholders with different power/interest statuses (e.g., adults that “are and are not the bill payers” at home). With the help of the grid, they could also “fine-tune” stakeholder mapping with respect to their perceived power/interest statuses. It is worth paying attention that, in the school environment, “teachers” were considered to have more power than the “government” and more interest in the issue than the “Department of Education.” This demonstrates a sometimes misplaced perception regarding the level of autonomy of individual schools. The ENERGE Committee members identified that the power/interest boundaries between some stakeholders could be fuzzy (e.g., architects/designers for schools). They also recognized that stakeholder mapping with respect to power/interest status in the system is an intricate task, which depends on multiple considerations and viewpoints.

In addition to its conceptual value, the exercise has a practical value. Usually, “crowd” stakeholders are monitored on a regular basis; “subjects” need to be completely informed; “context setters” must be kept satisfied by anticipating their needs, while “players” need to be thoroughly managed. If, for instance, the interest and power status of stakeholders is unclear, this may impede interventions within the project. In this case, effective tools, such as communication, commitments, social norms, or social diffusion, may not work effectively. For example, commitment (i.e., getting a verbal or written pledge to perform some behavior(s)) is directly related to a person’s interest (McKenzie-Mohr, 2020). If a person is not interested, it may not be efficient to heavily engage resources in commitment—particularly if they also lack power. Therefore, knowledge of the power/interest status of stakeholders helps to fine-tune related strategies in the project realization.

**Fig. 6:**
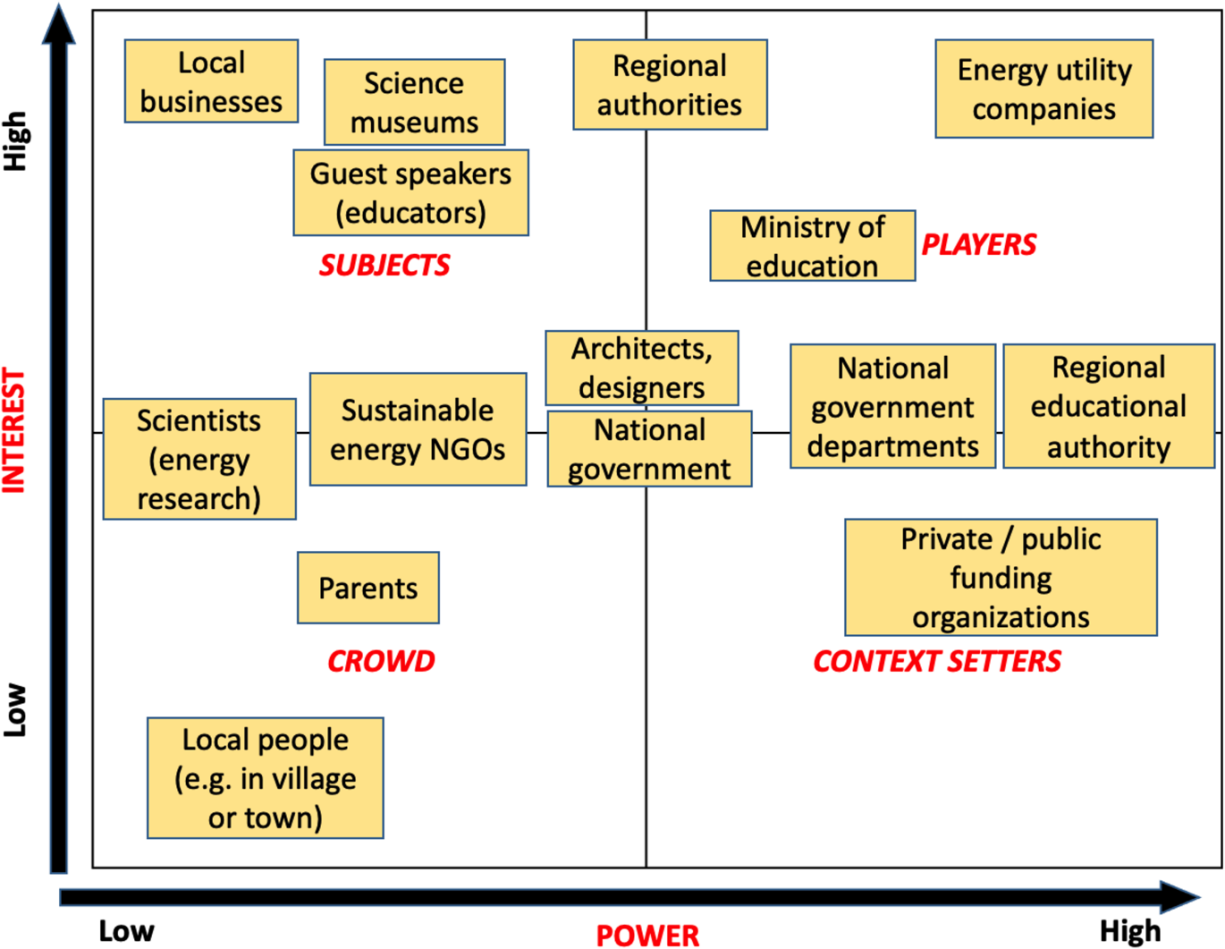
Power-vs-interest mapping of non-school stakeholders related to energy use in schools by the project partners

Another case of power-vs-interest grid exercise methodology application for the analysis of non-school stakeholders pertinent to energy use in the project schools was among the project partners, who represented various project countries. Figure 6 shows the results of mapping such stakeholders by the project partners.

This resulted in several important conclusions about the non-school stakeholders. Firstly, project partners, like the students in the previous exercise, were able to see a variability inside bigger stakeholder groups, thus segmenting larger groups of stakeholders into separate stakeholders with different power/interest statuses. Secondly, the project partners could adjust mapping stakeholders with respect to their perceived power/interest statuses and in relation to one another. They managed to see that some stakeholders could hold a transboundary position on the grid, which complicated the assigning of stakeholders to specific categories. Thirdly, another important result was that the project partners revealed the presence of inter-regional variability in assessing stakeholders. It became clear that the positions of some stakeholders with respect to their power and interest in the focal issue may vary, subject to a specific project country. Some stakeholders may be present in certain countries, but may not operate in other countries, while different stakeholders may perform identical or similar functions in various countries. For instance, regional educational authorities, which are strong in France and Northern Ireland, are not as prominent in Ireland, where their functions are managed either on the central or local level. This may pose a certain challenge for formalization of key stakeholders within the ENERGE project. It was also recommended to (a) establish a participation group of diverse stakeholders, which could broadly represent the focal issue to further analyze stakeholders, and (b) rely on a viable methodology for further stakeholder assessment. The latter could include such methods as focus groups (Morgan, 1998), value network analysis (Allee, 2008), and other methodologies.

### Stakeholder analysis survey

**Fig. 7:**
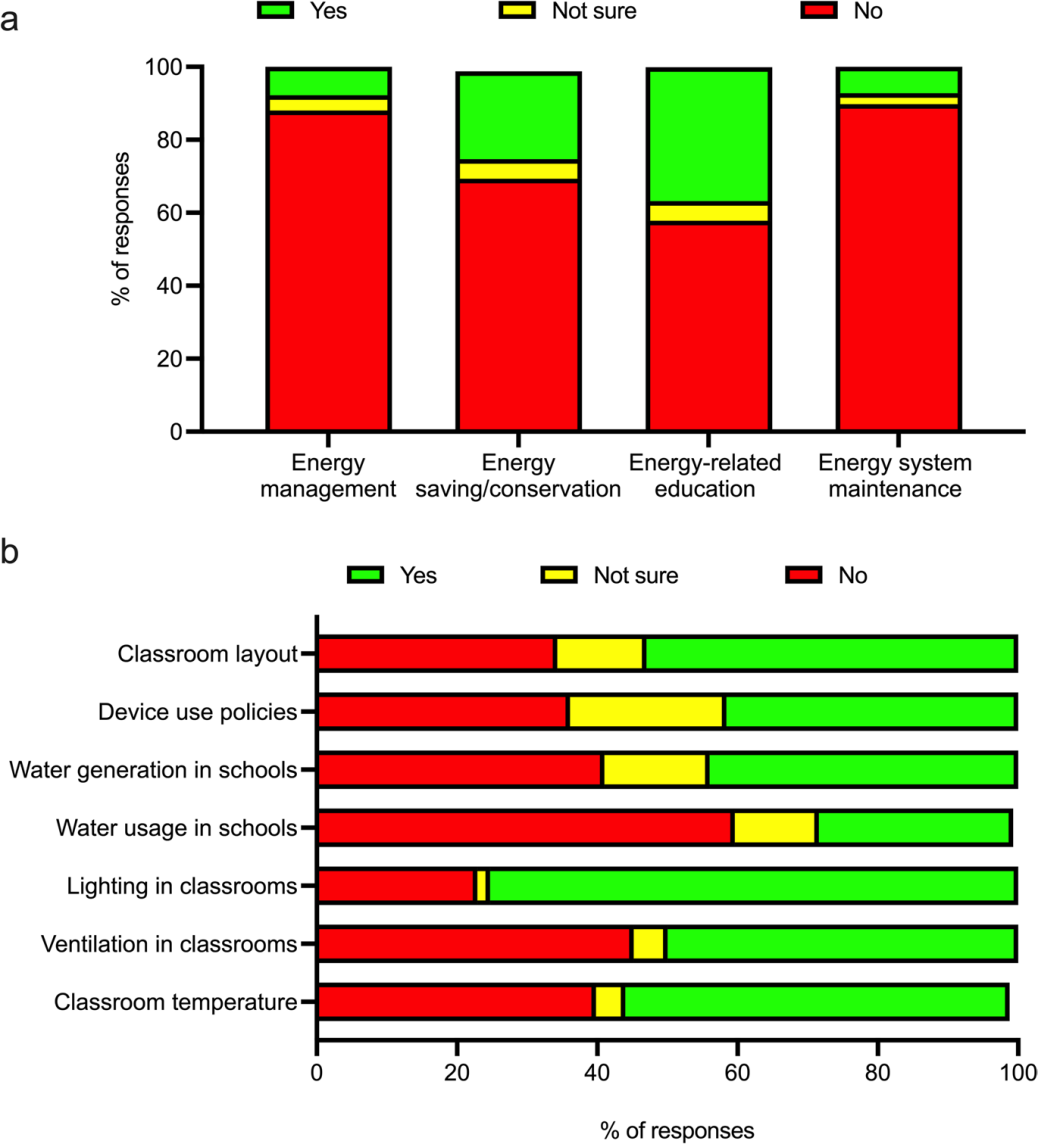
Stakeholder analysis survey results: (**a**) engagement of school personnel in energy-related activities (question: “Are you engaged in any of the following energy-related activities within the school?”) and (**b**) perception of control (locus of control) with respect to some comfort, environmental, and school operation issues (question: “In your opinion, do you have an opportunity to change or influence any of the following in school?”)

The implications of the stakeholder assessment survey for further research are multifaceted. The survey generated data for several important areas of school-based stakeholder interactions with energy. Figure 7a highlights the relation of school personnel to energy-related activities by answering the following question: “Are you engaged in any of the following energy-related activities within the school?” It is of interest that a modest number of the respondents deal with energy saving and conservation (42 responses out of 166, which make up less than 30% of the respondents). However, a larger amount of school personnel (about 40%) are engaged in energy-related education.

Figure 7b shows how respondents perceive the level of their control (locus of control) over some comfort, environmental, and school operation issues by answering the following question: “In your opinion, do you have an opportunity to change or influence any of the following in school?” The highest level of control is perceived to be over lighting in classrooms (125 positive responses out of 166, which account for about 75%), while the lowest level of control is over water usage in schools (47 positive responses out of 166, which account for less than 30%). In general, the level of control over other issues is not high and could average at around 50%.

The survey demonstrated how often the respondents performed concrete energy-related activities (question: “How often do you perform the following activities in school?”; Table 1). The highest level of regularity in performance of the activities is shown in activity 5 (“Turn off the lights when I leave the classroom or other school room, which won’t be occupied after me”). The lowest level of performance is demonstrated in relation to activity 4 (“Ask other experts to tell me more about energy and how to save it”). As alluded to before, it is known that those who engage in self-reporting tend to over-report environmental-conscious behavior. Different literature sources and case studies confirm that the level of over-reporting could be by several orders of magnitude (McKenzie-Mohr, 2020).

**Table 1:** Frequency of performance of energy-related activities by the respondents

Activity	Regularly	Occasionally	Never
1. Consider environmental impacts when making choices (e.g., consider environmental impact when you dispose of your waste)	66.9%	28.3%	4.8%
2. Learn more about energy and its use	25.9%	57.8%	16.3%
3. Learn more about how to save energy	21%	65.1%	13.9%
4. Ask other experts to tell me more about energy and how to save it	7.2%	57.2%	35.6%
5. Turn off the lights when I leave the classroom or other school room, which won’t be occupied after me	97.6%	2%	0.4%
6. Prevent heat loss by closing doors in cold times of the year	84.9%	13.3%	1.8%
7. Switch off overhead electrical lighting when there is sufficient daylight in the classroom or other school room	77.7%	18.1%	4.2%
8. Turn off electrical appliances/equipment when not in use to prevent electricity waste from a standby mode	65.7%	29.5%	4.8%

The data on stakeholder duties, energy-related activities, relevant communication patterns, and loci of control (with respect to energy use) suggest that school personnel could be roughly subdivided into the following four distinct (though possibly intersecting) categories:

1. School personnel that show a high degree of interest and activity in energy use and saving, which might be regular learners about energy saving and those asking other experts to tell them more about energy and its saving. The latter might be related to their professional jobs in school energy system maintenance and management.
2. Casual (routine) and regular performers of energy-saving activities. These activities might be small in effect but quite regular (both conscious and unconscious). Of course, as it was stated before, the regularity of performing these activities might be over-stated and affected by other factors.
3. Occasional performers of the energy-saving activities. This group might be prospective for energy efficiency–targeted projects. It is worthwhile to elucidate which factors block the school personnel from performing these activities regularly and then to address these factors via interventions.
4. Non-performers. There is a cohort of the school population that is not interested in energy use/saving and is not engaged in energy-saving activities. The important questions are why this negligence and non-performance happen and what can be done to tackle them.

### Energy literacy

**Fig. 8:**
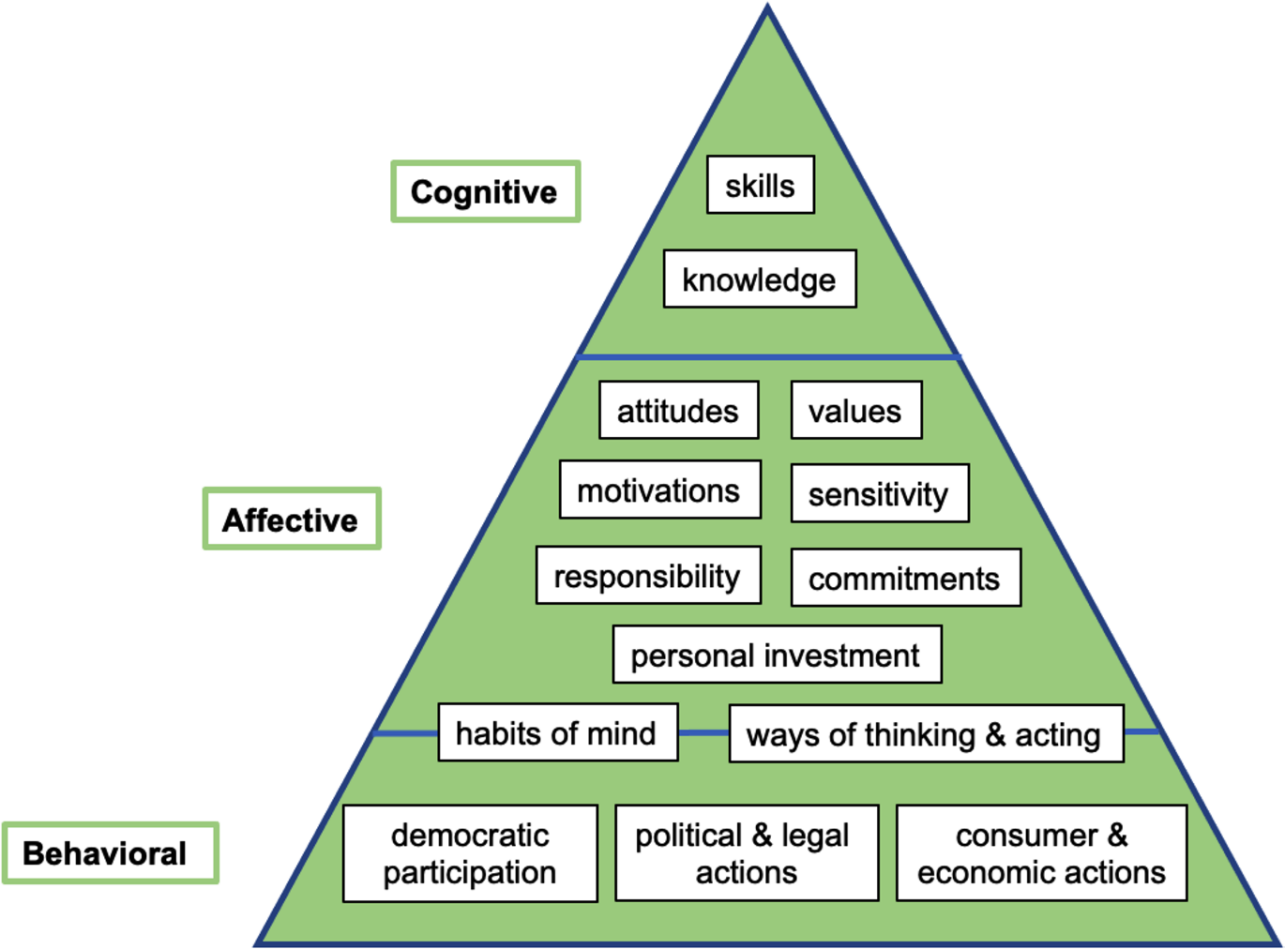
ENERGE energy literacy model

Within the ENERGE project, energy literacy encompasses not only the cognitive domain, but also the affective and behavioral characteristics—enabling citizens to think critically, solve problems, and make informed decisions about energy (Solomon, 1992). These three domains make up the essence of the ENERGE energy literacy model (Fig. 8).

**Fig. 9:**
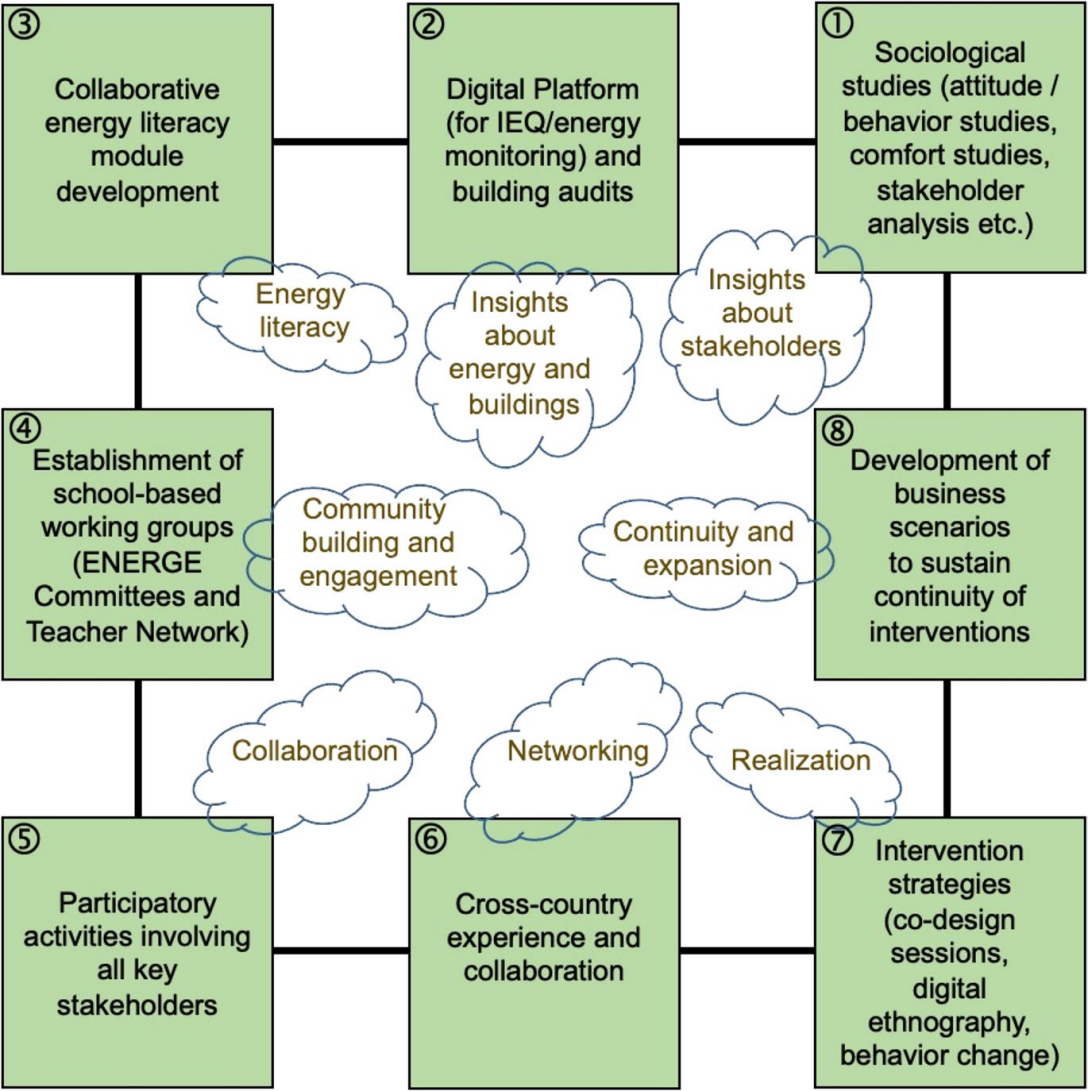
The ENERGE framework of energy efficiency in schools

Since the development of the elements of the model shown on Fig. 9 requires enduring efforts, the implementation of energy literacy modules, to supplement existing school curricula for students aged 12–18, can contribute primarily to the long-term impacts. ENERGE offers practical and affordable energy and building climate-related educational materials that are compliant with STEM curricula. This allows teachers to educate their students in science and technology–related topics using real-life data from the school building and its context, allowing for a more engaging and less abstract learning experience. At the same time, the students’ experiments in energy efficiency and indoor climate improvement strategies can result in a decrease in the energy use of the school and can allow school management to make evidence-based decisions on investments on energy efficiency measures. Therefore, the ENERGE project positions itself between a tool of building energy management and a resource of educational materials, thus bridging both domains.

Initial piloting of the ENERGE literacy modules, units, and activities in the project schools resulted in several recommendations. Firstly, it was recommended to broaden the scope of the ENERGE educational materials to address sustainability and energy and include related topics like water, waste, or circular economy. The interviews with the project school principals and teachers highlighted that there was a preference for implementing interdisciplinary STEM-related topics. Secondly, educational materials need to be matched with the skills and competences of each specific age group. Not only age but also education level needs to be considered when designing the ENERGE educational propositions. For instance, general and vocational education address different student learning outcomes. Moreover, the ENERGE educational materials need to align with national curriculum. In the targeted countries, two types of curricula are evident, the common core curriculum and differentiated curriculum. Thirdly, it is important that appropriate support is available for teacher professional learning to design and implement these educational materials, as teachers are key influencers on student engagement and learning.

### Key informant interviews with school management boards/administration

Interviews with the project school management boards/administration and teachers were instrumental in generating important insights. The question “Have you currently implemented energy efficiency solutions at your school(s)?” demonstrated the lack of systemic and consistent approaches to energy efficiency. Schools highlighted activities such as infrastructural changes (e.g., new windows, LED lights), the establishment of green/eco teams in the schools, and some behavioral change strategies (e.g., using energy-saving prompts, PCs in standby mode, visiting energy management training by administration officials, etc.). One of the project schools pointed to the controversy between the need to implement energy-saving steps (“plans to set up the computers to turn off automatically”) and the inconvenience of such practices (“teachers said they [PCs] took too long to start up in the mornings”).

The answers to the question “Who decides on taking energy efficiency measures and the selection of providers/installers?” confirmed the importance of senior administration of the schools, i.e., the board of management and principals, at the local (school) level. However, the decisive roles were played by the government or national education authorities. The question about 5-year expenditures on energy efficiency measures revealed that the activities directly related to energy saving (e.g., new window installations) were often funded on the basis of health and safety needs, rather than energy saving.

A panorama of responses was obtained when the interviewees answered the question “Do you currently use educational materials to educate students on energy, energy efficiency and/or sustainability? If yes, what materials do you use and where did you obtain them?” Firstly, such educational materials are based on curriculum, but are often developed by the teachers themselves. Secondly, there was an evident shortage of such materials and some issues with resourcing them. Thirdly, there can be a student contribution in the preparation of these materials. Finally, the specific focus on energy efficiency seems either rare or non-existent; the materials mostly cover general topics, like energy use, renewables, and sustainability. There is good potential for the ENERGE to provide educational materials to improve energy literacy in schools.

When reacting to different statements in the questionnaire, the interviewees provided valuable feedback. There a definite lack of clear-cut strategies and the required knowledge of formulating such strategies for making schools carbon neutral in the foreseeable future. All interviewees unanimously confirm the absence of sufficient funding to make school buildings healthy and energy neutral as they “have to fight for everything” and experience a “huge gap” with financing. The process of funding was described in the following ways: “It is always challenging to get funding for anything. Projects need to have a short payback and save money in order to have a better chance of getting funded.” and “Seeking funding takes a lot of work, the schools need to ‘play the game’ and chase the source of funding.” Such experience was not surprising, since, according to Eurostat data, in the period 2010–2019, the average general government expenditure on education as a ratio to GDP follows a decreasing trend (Eurostat 2022). While answering the question “School buildings should be used as living labs for teaching students on water, energy, waste etc.”, the interviewees marked the difficulty of getting student buy-in for such approaches. The vital role of family and parents was highlighted since the school cannot drive this transition to carbon neutrality alone.

Important results were generated when the interviewees were asked to assess preferable outcomes from the ENERGE project. The interviewees marked the importance of both key components of ENERGE, i.e., energy literacy education and data-based energy management. On the one hand, its educational component is paramount. On the other hand, ENERGE can give an opportunity “to get real-time insight in the energy performance and comfort of their school building, enabling evidence-based decision making on energy efficiency measures and tracking the impact of implemented measures.” However, it all depends on the practicality of ENERGE in both components as the educational component should produce important knowledge and skills, while the energy management component should make real savings and facilitate energy monitoring. As such, the interviewees suggest to “market” ENERGE as a holistic, all-in-one and continuous approach. Moreover, among some interviewees, there is a perception of an energy efficiency approach as a top-down, technology-driven, and totally deterministic tool where the role of schools is passive and active engagement of school building occupants is not pivotal, which is also governed by tight budgets of the schools. As one of the interviewees remarked: “…energy-efficiency plans need to be integrated into the school, not bolted on. It should be done by teachers in a bottom up approach from the students right up to the management level.”

The interviewees confirmed the vitality of stakeholders in energy efficiency, in general, and in the project realization, in particular:

You need to have the right person on the ground to drive it. You need someone that is naturally motivated to put in the effort to motivate others, otherwise it probably wouldn’t get used. Also, some of the teachers feel that they do not know enough to be educating the students on energy efficiency. They would need training on energy efficiency and sustainability in order to feel comfortable enough to work with the students and answer their questions. A network of teachers would be useful so that teachers from different schools can talk to each other and offer tips and tricks for engaging with the students.

To this extent, building relationships between various stakeholders, for example, between teachers and students in different countries or between schools and universities, is paramount.

### ENERGE energy efficiency framework for schools

Figure 9 visualizes the ENERGE energy efficiency framework for schools. The ENERGE framework builds on the above methodological and research contributions and consists of the eight key elements, each of which can generate vital outcomes (“clouds” on Fig. 9). Each of these elements implies a set of methods, tools, and techniques aimed at directly or indirectly influencing energy efficiency in schools. All elements are interlinked. For instance, the proprietary energy- and IEQ-monitoring ENERGE Digital Platform provides actionable insights about energy and buildings, but it can also be used to raise energy literacy by engaging students in energy-monitoring exercises, so it has a pedagogical as well as a practical value.

It is pertinent to single out several important interlinking features that distinguish this framework from other approaches described in the literature review. Firstly, the framework strongly relies on sociological studies, especially on different types of stakeholder analysis (box 1, Fig. 9), which should become an indispensable part of approaching the issue of raising energy efficiency in schools. Secondly, there is a significant focus on participation, collaboration, engagement, empowerment, and community building, including on a cross-country basis (boxes 4 and 5, Fig. 9). Moreover, this collaboration permeates all elements of the framework, including the Digital Platform operation (box 2, Fig. 3) and energy literacy development (box 3, Fig. 9). The framework confirms that energy efficiency is not only a technological challenge, but also a personal and social issue requiring a thorough understanding of various characteristics of relevant stakeholders and engaging them in collaborative efforts. Thirdly, special care is given to raising energy literacy, which demonstrates rigor, consistency, and active consultations with various stakeholders. The elements on intervention strategies (box 8, Fig. 9) and business scenario development are beyond the scope of this publication as the ENERGE project is still in progress, while the stages of interventions and post-project maintenance will be reported separately due to the large volume of generated materials and longitudinal character of these elements.

## Discussion

Energy literacy, which is a cornerstone of the ENERGE framework, is known to correlate with such constructs as knowledge, attitude, self-efficacy, and behavior (Chen et al., 2015). Likewise, the developed ENERGE energy literacy model comprises affective, cognitive, and behavioral aspects. The strength of the framework lies in its holistic approach to energy literacy module development, which considered all these aspects to present a balanced mix of theory, activities, exercises, curriculum-supporting materials, and direct energy efficiency measures.

Another strength of the ENERGE framework in the domain of energy literacy is in its collaborative approach to the development of curriculum-based energy literacy modules and other pedagogical tools, which were a manifestation of international collaborative efforts. In this case, collaboration can become a way of combatting “uncertainties about instructional content” (Erçetin et al., 2015, p. 150), lack of interdisciplinarity and associated limitations of standardized curricula. However, it should be noted that energy literacy is a complex, multi-stage and multi-dimensional educational and learning process, dependent on the interplay of various sociodemographic, psychographic, cognitive, cultural, and personal factors (Chen et al., 2015). The viability of any approach in the domain of energy literacy requires significant practical efforts, which should demonstrate consistency over many years and across different contexts.

Technology underlies the whole spectrum of technological and innovative responses to energy efficiency, such as devices, materials, information inputs, energy, ideas, methods, etc. The use of different digital devices in schools—particularly smart meters—could lead to significant benefits, including in customization of energy-saving advice and provision of actionable insights. To this extent, the installation and operation of the IEQ- and energy-monitoring digital platform in schools is another key element of the ENERGE framework. Apart from technological prowess, this platform can be used for energy literacy purposes and empowerment of school stakeholders to make actionable and informed decisions on the basis of the generated data. However, the actionability of these insights is predicated on multiple factors including accuracy and usefulness of data, user friendliness of interfaces, and deep embeddedness of technological solutions into the fabric of specific built environment and its operational practices (Kimura et al., 2018). Any practical user of such devices in a complex dynamic environment such as a school should fully appreciate the complex interactions between generated data and specifics of daily routines (Sahakian et al., 2021). As with energy literacy, robust testing of digital automation in schools in real-world settings is paramount to ensure the responsiveness of building users to energy efficiency-raising measures is understood (Hoicka & Parker, 2018; Kimura et al., 2018).

While the ENERGE framework does not develop innovative technologies per se, its potential lies within the combination of existing technologies, improved methodologies, and novel ideas to provide a holistic response to the issue of energy efficiency. Importantly, “technology in learning environments should be easy to use, reliable, and pedagogically relevant” and should “promote collaboration, engagement, interaction or deeper learning” (Casanova et al., 2020, p. 414). This is what defines the essence of the digital platform within the ENERGE framework.

Technology use is inextricably linked to values, perceptions, norms, and other sociopsychological aspects (Guerreiro et al., 2015). Therefore, with respect to energy systems, Longhurst and Chilvers (2019, p. 973) ascertain that “what is often presented as a primarily ‘technical’ transition [of energy systems] is always normative in bringing forward particular forms of social and political order.” In connection with this, Wittmayer et al. (2022) stress the role of societal and cultural values, norms, symbols, feelings, emotions, perceptions, and rituals. Likewise, the ENERGE framework implies value co-creation/exchange between various stakeholders across all ENERGE dimensions. There is now a consensus that the long-term task of improving energy efficiency in buildings is untenable without consistent and collaborative efforts of all stakeholders within various economic, political, socio-cultural, technological, pedagogical, personal, and other dimensions and systems (Pietrapertosa et al., 2021). A problem of responsible energy use in schools is shaped by a myriad of such stakeholder interactions, which include “a vast kaleidoscope of simple and complex relational exchanges” (Hastings & Domegan, 2018, p. 71). The ENERGE project has provided links between the schools and the local neighborhood. This has involved engaging with local public attractions like museums and libraries (where demonstration versions of the ENERGE platform have been presented) along with local authorities, regional authorities, and even a multinational company. Several local and regional authorities are part of the ENERGE consortium, which has helped build these links even stronger.

Therefore, the ENERGE framework focuses on stakeholder collaboration (between individuals, groups, and entities), networking, community building, and getting insights about the stakeholders. However, the level of multidimensionality and systematicity of this task is significantly underrepresented and misunderstood; the suggested framework, visualized with the help of Fig. 9, is aimed to raise this level.

Finally, the ENERGE framework builds upon cross-country experience and collaborations, linking students and school staff from different countries in sharing their experience, knowledge, lived stories, etc., that are related to energy use and raising energy efficiency. Cross-cultural aspects are known to shape differences in energy conservation behaviors within and across various countries (Goggins et al., 2022; Long et al., 2018). Despite these differences, ENERGE helps to find a common ground and generalized approaches to tackling diverse issues of energy efficiency.

## Limitations and coping strategies

The aim of this research was to present a systemic framework to facilitate energy reduction in schools across different European contexts. However, the implementation of this task encountered several challenges, and thus, it has certain limitations.

Like any methodology, the ENERGE framework and methodology will adapt given further feedback from new participating schools. It should also be mentioned that its implementation coincided with Covid where schools were often shut for prolonged periods. Establishing baselines for energy consumption was more challenging, and subsequent to Covid, many schools operated with windows open in winter for ventilation purposes. The health context was obviously more important than energy considerations at that time. However, despite this, the ENERGE framework showed its robustness by allowing schools to engage with education materials, surveys, data via the digital platform, and other ENERGE options. We consider the results and framework very relevant given the lack of such systemic frameworks, which could be readily adopted by schools. This is even more important in the light of the current energy cost rises, where significant and immediate reductions in energy consumption, which will affect public institutions, including schools, are required in the EU. To this extent, the wide range of methodological tools that we tested and are present in the format of multiple resources (e.g., ready-made energy literacy modules, the ENERGE digital platform, stakeholder analysis exercises, etc.) give schools a variety of practical methods to positively impact energy education and efficiency in their premises. The ENERGE-based approach increases certainty about “what works” and advocates the need to use a strategic and long-term perspective, when the results of interventions are evaluated on a longitudinal and multi-dimensional basis. ENERGE shows how the outcomes of energy efficiency can go well beyond reducing energy consumption by a certain percentage value. It is considered of greater importance to first create a systemic framework for realization of various energy-related initiatives and strategies. Without such a framework, any intervention in the domain of energy efficiency has a strong chance of becoming disconnected from existing theories and the school cohort and becoming a series of one-off events.

There is a seemingly non-homogenous mode of the framework application in the project schools in the different countries. The Covid pandemic was a serious impediment for carrying out the framework application in a more homogenous way. However, the generalized framework was not intended to be homogeneously manifested or even tested in each country. At the early stages of the project, the project partners carried out extensive research of the variations among the national educational systems in each of the project countries. Besides, the ENERGE project schools involve different types of educational establishments, like city gymnasiums, vocational colleges, small (almost rural) schools, etc. All these schools feature different governance models and diverse approaches to energy management. In general, the ENERGE project elucidated this large diversity of educational systems in different countries and of specific educational establishments even in one country. This showed a very rich and vibrant picture of the context within which schools try to raise or maintain energy efficiency. The ENERGE project thus demonstrates that successful energy management frameworks need to be aware of these contexts. Apart from that, all the ENERGE project partners were actively involved in contacting and collaborating with the schools’ staff and students, as well as in the operation of the ENERGE committees, which facilitated seeing unique circumstances of energy management in each specific school and creating what might be characterized as the ENERGE learning community. This community building is in progress and is supported by the installed ENERGE digital platform.

Therefore, the differences in national education systems and in specific schools are too large for “homogenous” implementation of any energy-saving platform. Any energy-saving framework should be adapted for concrete conditions. This goes in line with the concept of contingent sustainability, which suggests that sustainable interventions should be determined specifically for each location and culture through public participation and stakeholder negotiations (Morelli et al., 2013).

## Conclusions

This paper provides a systemic framework for energy efficiency in schools, aimed at the transformation of thirteen project schools into more energy-responsible places. Since energy efficiency in the educational environment is a very complex issue, exacerbated by a range of interrelated personal, technological, social, and other factors, addressing this issue requires a systemic approach. Despite some available theoretical and practical endeavors undertaken to raise the level of systematicity in this domain, the shortage of systemic energy efficiency approaches is currently a significant gap. The suggested ENERGE framework addresses this situation and offers a range of tested methodological tools, which have already generated important results. The contribution of this paper lies in demonstrating how a systemic approach to energy efficiency can benefit schools.

The benefits of the suggested framework over other approaches are numerous. Its holism expands to practically all domains of energy management in schools. Curriculum-embedded energy literacy education is aimed to provide comprehensive knowledge and skills about the focal issue. Digital transformation of the school energy sector monitors IEQ/energy and simultaneously serves educational purposes. Deep reliance on sociological studies caters for an important mission of knowing key stakeholders (at micro, meso, and macro levels), which can help to fine-tune relevant behavior change interventions to target specific segments of the addressed audience. Significant attention is given to the development of viable business scenarios for future manifestation of the framework and for the expansion of its experience to other schools. An indispensable element of the framework is in its sheer focus on collaboration, engagement, and participation of different stakeholders, including on a cross-country basis. The framework creates school-based groups of activists that operate as its ambassadors and conduits of the generated knowledge and skills. Everything is performed collaboratively. For instance, energy literacy modules are developed via an extensive process of collaboration. The level of interconnectivity of the elements of the framework is extremely high, meaning that each element is related to others and all elements are mutually beneficial. The framework is backed up by a comprehensive and well-developed strategy of its realization, which hinges on such approaches as co-design sessions, digital ethnography, and behavior change interventions. Taking into consideration all these benefits, the ENERGE framework can arouse significant interest and deliver benefits among different stakeholders associated with the issue of energy management in schools.

## Data Availability

The datasets generated during and/or analysed during the current study are available from the corresponding author on reasonable request.
